# Eficacia de piezoeléctrico versus pieza de mano recta a baja velocidad dentro de la extracción quirúrgica de terceros molares inferiores retenidos

**DOI:** 10.21142/2523-2754-1004-2022-129

**Published:** 2023-12-26

**Authors:** Bryan Morales-Sambachi, Adriana Andrade-Peñafiel

**Affiliations:** 1 Facultad de Odontología de la Universidad Central del Ecuador, Quito, Ecuador. bryanml@hotmail.es, alandradep@uce.edu.ec Universidad Central del Ecuador Facultad de Odontología Universidad Central del Ecuador Quito Ecuador bryanml@hotmail.es alandradep@uce.edu.ec

**Keywords:** extracción dental, tercer molar, edema, dolor posoperatorio, trismo, tooth extraction, third molar, edema, pain postoperative, trismus

## Abstract

**Objetivo:**

Determinar la eficacia del piezoeléctrico vs. la pieza de mano recta a baja velocidad en cirugías de terceros molares inferiores retenidos, en relación con los efectos posquirúrgicos (edema, dolor y trismus) y el tiempo operatorio de osteotomía, en pacientes del Servicio de Cirugía Maxilofacial del Hospital de Especialidades Fuerzas Armadas N.° 1.

**Método:**

Estudio clínico observacional, analítico, longitudinal de cohorte prospectivo. Se tomó una muestra de 40 pacientes de ambos sexos con edades entre 18 y 25 años, divididos en dos grupos de 20 pacientes cada uno. En el grupo A se realizaron las osteotomías con piezoeléctrico y en el grupo B, con pieza de mano recta rotatoria a baja velocidad. El edema se midió con el método de Amin y Laskin; el dolor, con la Escala Visual Análoga (EVA); y la apertura bucal, con el instrumento pie de rey, al tercer, quinto y octavo día.

**Resultados:**

En el grupo A, el edema (11,72 mm), dolor (4,67) y apertura bucal (39,49 mm) fueron menores que en el B: edema (12,29 mm), dolor (4,8) y apertura bucal (35,83 mm), mientras que el tiempo de osteotomía fue significativamente mayor con el piezoeléctrico (6,54± 3,05 min) en comparación con la pieza de mano recta (1,55 ± 0,57 min). Existió diferencia del edema, dolor y trismus en condiciones normales, en contraste con lo obtenido a los 3, 5 y 8 días entre los dos instrumentos.

**Conclusión:**

Los efectos posquirúrgicos, como edema, dolor y trismus de la exodoncia de los terceros molares inferiores retenidos, se presentan con menor intensidad con el piezoeléctrico, en comparación con la pieza de mano recta, por lo que se recomienda el uso del primero.

## Abstract (en)

**Objective:**

Determine the effectiveness of the piezoelectric handpiece vs. the straight handpiece at low speed in surgeries of impacted lower third molars, in relation to post-surgical effects (edema, pain and trismus) and the operative time of the osteotomy, in patients attending the Maxillofacial Surgery Service of the “Hospital de Especialidades Fuerzas Armadas N°1”.

**Method:**

It is an observational, analytical, longitudinal prospective cohort clinical study. A sample of 40 patients of both sexes aged between 18 and 25 years was taken, divided into two groups of 20 patients each, in group A extractions were performed with piezoelectric and in group B with straight rotary handpiece at low velocity. The results were analyzed on the third, fifth and eighth day, the edema was measured with the method of Amin and Laskin, for the pain the Visual Analogous Scale (VAS) was used, and for the presence of trismus with the instrument foot of king.

**Results:**

In group A, edema (11.72 mm), pain (4.67) and mouth opening (39.49 mm) were lower than in group B edema (12.29 mm), pain (4.8) and mouth opening (35.83 mm) and osteotomy time was significantly longer with piezoelectric (6.54 ± 3.05 min) compared to the straight handpiece (1.55 ± 0.57 mm). min) and there was a difference in edema, pain and trismus under normal conditions in contrast to what was obtained at 3, 5, 8 days between the two instruments.

**Conclusion:**

the post-surgical effects of edema, pain and trismus of the extraction of retained lower third molars are less intense with the piezoelectric compared to the straight handpiece, the use of the piezoelectric being recommended.

## INTRODUCCIÓN

Los terceros molares inferiores retenidos pueden causar problemas en la región orofacial, incluida la caries dental, con los consecuentes abscesos y bolsas periodontales en segundos molares, apiñamientos, periocoronitis y alteraciones de la articulación temporomandibular. Por ello, algunos especialistas recomiendan su extracción, lo que involucra un procedimiento quirúrgico con técnicas, idealmente, mínimamente invasivas ^1^.

Entre los métodos más empleados para la extracción de los terceros molares destaca el uso de instrumentos rotatorios, cuyas desventajas son la producción de macrovibraciones que causan incomodidad al paciente u operador ^3^, posibles daños en los tejidos adyacentes, sobrecalentamiento óseo e, incluso, consecuencias posoperatorias más traumáticas como el aumento del edema, dolor y trismus en el paciente ^2^. 

Entre las complicaciones más frecuentes en la extracción de terceros molares retenidos se reporta el edema orofacial, que es la inflamación debido a la acumulación de líquido en los tejidos. Se distingue por el aumento de la dilatación vascular y la permeabilidad tisular ^6^. Fue identificado través del método de Amin y Laskin. El dolor es otra molestia común, como respuesta del sistema nervioso después de la extracción del órgano dental, y depende de la percepción de cada individuo ^7^; en el presente estudio se estableció utilizando la escala visual análoga (EVA). Finalmente, el trismus suele presentarse como espasmos de los músculos masticatorios y dificultad para la apertura y cierre de la cavidad oral ^8^, por lo cual utilizamos el instrumento pie de rey.

Considerando estos factores, aparece el piezoeléctrico como método alternativo que se basa en microvibraciones a frecuencias ultrasónicas de 24 a 36 kHz, menos invasivas para realizar un corte óseo eficiente, con mayor nivel de precisión, seguridad, menor daño tisular y mejor visibilidad durante el procedimiento de corte y durante la extracción de terceros molares, en comparación con la pieza de mano recta ^1^^,^^4^. Sin embargo, involucra un mayor tiempo de intervención quirúrgica ^5^. 

Esta investigación tiene como objetivo determinar la eficacia de piezoeléctrico versus la pieza de mano recta a baja velocidad, tanto en los efectos posquirúrgicos (edema, dolor y trismus) como en el tiempo de osteotomía, respecto de la extracción de terceros molares inferiores retenidos en pacientes que acuden al área de Cirugía Maxilofacial del Hospital de Especialidades Fuerzas Armadas N.° 1. La información obtenida a través de la presente investigación contribuye al conocimiento científico de los estomatólogos y permite el uso del piezoeléctrico en cirugía bucal, lo que logra reducir los efectos propios posquirúrgicos (edema, dolor y trismus) en la exodoncia de terceros molares retenidos y una pronta recuperación del paciente, para que pueda incorporarse a sus actividades cotidianas de forma rápida y segura.

## MATERIALES Y MÉTODOS

Es un estudio clínico observacional, analítico, longitu-dinal de cohorte prospectivo, que cuenta con la aprobación del Subcomité de Ética de Investigación IE02-154-2021-CEISH-Universidad San Francisco de Quito. La población estuvo conformada por pacientes de ambos sexos entre los 18 y 25 años que acudieron al Hospital de Especialidades Fuerzas Armadas N.° 1, entre agosto y octubre de 2021, para realizarse la extracción de los terceros molares inferiores. La muestra del estudio fue no probabilística a conveniencia según el artículo base ^1^. Se contactó a 40 pacientes a quienes se les explicó el procedimiento a realizar, firmaron el consentimiento informado y cumplieron con los criterios de selección establecidos para el estudio. 

Se incluyó a pacientes que requirieron extracción quirúrgica de terceros molares inferiores (38,48) ASA I sin infecciones bacterianas o agudas como pericoronaritis, absceso alveolar agudo o fibrosis oral submucosa, cordales según la clasificación de Winter; mesioangulado y Gregory y Pell; tipo B clase I y II acorde del nivel de complejidad, además de que podían ser intervenidas tanto por piezoeléctrico como por pieza de mano recta a baja velocidad. Se excluyó a los pacientes con compromiso sistémico.

Los investigadores informaron el procedimiento a los pacientes de una manera clara, comprensible y sencilla, incluidos los pasos, la sintomatología posterior y las posibles complicaciones. Posteriormente, firmaron el consentimiento informado. Para su análisis, se utilizó el programa Microsoft Excel Versión 2204, en el cual se generó un sistema de identificación para cada participante mediante la primera letra del segundo nombre, la primera letra del primer apellido y los dos últimos dígitos de la cédula de ciudadanía, la edad y el sexo, asegurando su anonimato.

Los pacientes fueron atendidos por el cirujano según la cita asignada por el hospital. En el momento que acudía el paciente, recibía el tratamiento según la disponibilidad de equipos para realizar la osteotomía al momento de su atención. Un total de 20 pacientes fueron atendidos con piezoeléctrico y 20 con una pieza de mano recta a baja velocidad.

Para la cirugía, el investigador fue previamente calibrado por la tutora en las mediciones del edema, dolor y trismus posquirúrgico, para lo cual se realizó una prueba Kappa cuyo valor fue mayor a 0,8, lo que implica una concordancia aceptable entre las medidas del tutor y el investigador.

### 2.1. Técnica quirúrgica

En el grupo A, el instrumento utilizado fue el piezoeléctrico marca Woodpecker (Sirgic Touch Led, 3RD generation, Guangxi-China), a una frecuencia de 24 KHz-36 KHz, con una fresa chapada en oro N.° US1. En el grupo B se utilizó la pieza de mano recta rotatoria a baja velocidad neumática marca NSK (Kanuma, Japón), a una velocidad entre 0 y 40 000 rpm, y una fresa de carburo tungsteno redonda N.° 8.

### 2.2. Procedimientos clínicos

El día de la cita asignado por el hospital, el cirujano especialista, 15 minutos antes de la exodoncia, procedió a la toma de la apertura bucal de cada uno de los pacientes con el instrumento pie de rey. Se registró la medida desde el borde incisal de la pieza dental 1.1 y 4.1. El investigador anotó los datos en la hoja de recolección de información; luego, se procedió a señalar con un marcador estos puntos anatómicos DHS: tragus-pogonion, DHC: tragus-comisura bucal, DV: ángulo palpebral externo hasta el ángulo del gonion. Una vez señalados dichos puntos, fueron unidos con una regla flexible y el dato obtenido se registró en la hoja de recolección de datos. 

El cirujano especialista, cumpliendo todas las normas de asepsia y antisepsia, colocó lidocaína al 2% con adrenalina 1:80000. Para la sinéresis utilizó un bisturí mango #3 hoja #15, e hizo una incisión lineal vestibular intrasurcular desde la pieza dental 3.6 hasta la zona retromolar en la zona izquierda, y en la pieza dental 4.6 hasta la zona retromolar en la zona derecha. El levantamiento de colgajo lo hizo con el periostótomo de Molt. Para la osteotomía en la zona retromolar, se utilizó el piezoeléctrico a una frecuencia de 24 KHz-36 KHz, con una fresa chapada en oro N.° US1 o una pieza de mano recta a baja velocidad, entre 0 y 40 000 rpm, con una fresa de carburo tungsteno redonda N.° 8, con irrigación constante de suero fisiológico. Una vez visible la corona clínica del tercer molar, se procedió a la odontosección longitudinal utilizando una pieza de mano recta en ambos grupos y fresa 703 de Linderman, culminando la odontosección con elevador recto media caña mediano, exodoncia propiamente dicha, cuidados de la cavidad sinéresis con 3 puntos simples discontinuos con hilo se sutura nylon 3/0.

Durante la cirugía, el asistente del cirujano informó el comienzo y la finalización de la osteotomía con el instrumento piezoeléctrico y la pieza de mano recta a baja velocidad en cada paciente. Se lo midió con un cronómetro digital para el respectivo registro en la hoja de recolección de datos. 

Al tercer, quinto y octavo día, el investigador tomó las mediciones del edema con el método de Amin y Laskin; para el dolor se utilizó la Escala Visual Análoga (EVA), mientras que la determinación de la presencia de trismus se realizó con el instrumento pie de rey. La información obtenida se registró para su posterior análisis.

### 2.3. Análisis estadístico

Se realizó una estadística descriptiva (media, desviación estándar, porcentaje y frecuencia), las diferencias entre los efectos posquirúrgico edema, dolor y trismus con las variables dependientes, se realizó chi cuadrado y T Student a un nivel de significancia de p < 0,05 y nivel de confiabilidad del 95%.

## RESULTADOS

El mayor valor del edema, medido por el método de Amin y Laskin, se identifica a los 3 días como efecto de la extracción con la pieza de mano recta de baja velocidad (12,28 ± 0,53 mm), seguido por los 5 días (12,03 ± 0,47 mm); el menor valor fue el observado en el que usaron piezoeléctrico a los 8 días (11,37 ± 0,48 mm). Se identificó la existencia de una diferencia significativa al tercero (p = 0,001), quinto (p = 0,001) y al octavo (p = 0,01) día entre la pieza de mano recta rotatoria a baja velocidad y el piezoeléctrico (Tabla 1).

**Tabla 1:** Comparación entre los dos instrumentos según las variables edema, dolor y trismus

Variables	N	Piezas de mano recta rotatorio				Piezoeléctrico				p-valor
		Media	D. E.	Mín.	Máx.	Media	D. E.	Mín.	Máx.	
Edema										
Valor normal	20	11.39	0.46	10.60	12.50	11.25	0.41	10.60	12.16	0.340
Edema - 3 días	20	12.28	0.53	11.40	13.30	11.71	0.51	10.90	12.90	0.001
Edema - 5 días	20	12.03	0.47	11.30	12.80	11.52	0.51	10.80	12.80	0.001
Edema - 8 días	20	11.76	0.40	11.10	12.60	11.37	0.48	10.60	12.60	0.010
Dolor										
Dolor - 3 días	20	4.80	2.30	2.00	9.00	4.66	1.84	2.00	8.00	0.850
Dolor - 5 días	20	3.55	2.18	1.00	8.00	2.00	2.29	0.00	8.00	0.040
Dolor - 8 días	20	1.35	1.69	0.00	6.00	0.60	1.39	0.00	5.00	0.130
Trismus										
Valor normal	20	40.26	12.09	20.90	65.20	47.29	6.33	33.50	56.60	0.030
Trismus 3 días	20	33.24	13.28	4.70	57.30	33.03	7.89	17.20	45.60	0.950
Trismus 5 días	20	34.32	13.55	4.90	58.30	35.48	7.92	17.40	47.70	0.740
Trismus 8 días	20	35.83	13.49	10.00	60.40	39.49	6.60	24.50	49.80	0.280

N: número de pacientes. DE: desviación estándar. Mín.: mínimo. Máx.: máximo

Prueba T de Student (p < 0.05)

Los menores valores de dolor posquirúrgico evaluado por la escala visual análoga EVA se presenta con el piezoeléctrico a los 3 días (4,67 ± 1,85), 5 días (2 ± 2,29) y 8 días (0,6 ± 1,39), en comparación con la pieza de mano recta a baja velocidad. Solo existió diferencia significativa del dolor al quinto (p = 0,04) día entre los dos instrumentos (Tabla 1).

La mayor apertura bucal en milímetros con la ayuda del instrumento pie de rey se evidenció con el piezoeléctrico a los 5 (35,48 ± 7,92 mm) y 8 días (39,49 ± 6,61 mm). No existe diferencia significativa entre las dos técnicas al comparar el trismus al tercer, quinto y octavo día (p > 0,05) (Tabla 1).

Los resultados descriptivos demuestran que prevalece el grupo de 18 a 21 años, y se presenta el dolor al quinto ^(^p = 0,03) y octavo (p = 0,001) día. Por lo tanto, existe asociación entre el uso de la pieza de mano recta y la edad. El trismus se presenta al quinto (p = 0,02); por lo tanto, existe asociación entre el piezoeléctrico y el sexo. En los demás cruces del dolor, trismus y edema con la edad y el sexo no existió diferencia significativa (p > 0,05) (Tabla 2).

**Tabla 2:** Comparación entre los dos instrumentos y las variables edema, dolor y trismus, según la edad y sexo

Variables		Pieza de mano recta rotatorio			p-valor	Piezoeléctrico			p-valor
		Leve	Moderada	Grave		Leve	Moderada	Grave	
Edema 3 días									
Edad	18 a 21 años	4 (25%)	5 (31.3%)	7 (43.8%)	0.740	8 (57.1%)	4 (28.6%)	2 (14.3%)	0.460
	22 a 25 años	1 (25%)	2 (59.0%)	1 (25%)		5 (83.3%)	1 (16.7%)	0 (0%)	
Edema 5 días									
Edad	18 a 21 años	9 (56.3%)	4 (25%)	3 (18.8%)	0.160	11 (78.6%)	2 (14.3%)	1 (7.1%)	0.790
	22 a 25 años	1 (25%)	3 (75%)	0 (0%)		5 (83.3%)	1 (16.7%)	0 (0%)	
Edema 8 días									
Edad	18 a 21 años	12 (75%)	4 (25%)	0 (0%)	1.000	12 (85.7%)	2 (14.3%)	0 (0%)	0.320
	22 a 25 años	3 (75%)	1 (25%)	0 (0%)		6 (100%)	0 (0%)	0 (0%)	
Dolor 3 días									
Edad	18 a 21 años	9 (56.3%)	6 (37.5%)	1 (6.3%)	0.060	10 (71.4%)	4 (28.6%)	0 (0%)	0.270
	22 a 25 años	2 (50%)	0 (0%)	2 (50%)		4 (66.7%)	1 (16.7%)	1 (16.7%)	
Dolor 5 días									
Edad	18 a 21 años	13 (81.3%)	2 (12.5%)	1 (6.3%)	0.030	13 (92.9%)	0 (0%)	1 (7.1%)	0.240
	22 a 25 años	1 (25%)	3 (75%)	0 (0%)		5 (83.3%)	1 (16.7%)	0 (0%)	
Dolor 8 días									
Edad	18 a 21 años	16 (100%)	0 (0%)	0 (0%)	<0.001	14 (100%)	0 (0%)	0 (0%)	0.110
	22 a 25 años	2 (50%)	2 (50%)	0 (0%)		5 (83.3%)	1 (16.7%)	0 (0%)	
Edema 3 días									
Sexo	Masculino	4 (44.4%)	2 (22.2%)	3 (33.3%)	0.180	6 (60%)	4 (40%)	0 (0%)	0.140
	Femenino	1 (9.1%)	5 (45.5%)	5 (45.5%)		7 (70%)	1 (10%)	2 (20%)	
Edema 5 días									
Sexo	Masculino	5 (55.6%)	2 (22.2%)	2 (22.2%)	0.480	8 (80%)	2 (20%)	0 (0%)	0.510
	Femenino	5 (45.5%)	5 (45.5%)	1 (9.1%)		8 (80%)	1 (10%)	1 (10%)	
Edema 8 días									
Sexo	Masculino	6 (66.7%)	3 (33.3%)	0 (0%)	0.430	10 (100%)	0 (0%)	0 (0%)	0.130
	Femenino	9 (81.8%)	2 (18.2%)	0 (0%)		8 (80%)	2 (20%)	0 (0%)	
Trismus 3 días									
Sexo	Masculino	6 (66.7%)	3 (33.3%)	0 (0%)	0.490	9 (90%)	1 (10%)	0 (0%)	0.120
	Femenino	5 (45.5%)	5 (45.5%)	1 (9.1%)		6 (60%)	4 (40%)	0 (0%)	
Trismus 5 días									
Sexo	Masculino	7 (77.8%)	2 (22.2%)	0 (0%)	0.290	10 (100%)	0 (0%)	0 (0%)	0.020
	Femenino	5 (45.5%)	5 (45.5%)	1 (9.1%)		6 (60%)	4 (40%)	0 (0%)	
Trismus 8 días									
Sexo	Masculino	7 (77.8%)	2 (22.2%)	0 (0%)	0.450	10 (100%)	0 (0%)	0 (0%)	0.130
	Femenino	6 (54,5%)	4 (36,4%)	1 (9,1%)		8 (80%)	2 (20%)	0 (0%)	

Prueba Chi cuadrado (p < 0,05)

Se aplicó la prueba estadística T de Student y se verificó una diferencia significativa entre la media del edema y trismus en condiciones normales, en contraste con la obtenida al tercer, quinto y octavo día al utilizar la pieza de mano recta rotaria (p < 0,05); al igual que al comparar el dolor, trismus y edema posquirúrgico al tercer, quinto y octavo día (p < 0,05) (Tabla 3).

**Tabla 3:** Contraste de las variables iniciales y posquirúrgica con el uso de la pieza de mano recta

Variables	Media	D. E.	95% IC		t	gl	p-valor
			Mín.	Máx			
Edema - normal - Edema - 3 días	-0.90	0.45	-1.11	-0.69	-8.9	19	<0.001
Edema - normal - Edema - 5 días	-0.64	0.37	-0.82	-0.47	-7.72	19	<0.001
Edema - normal - Edema - 8 días	-0.37	0.25	-0.49	-0.26	-6.64	19	<0.001
Edema - 3 - Edema - 5 días	0.25	0.17	0.18	0.33	6.79	19	<0.001
Edema - 3 - Edema - 8 días	0.52	0.42	0.32	0.72	5.53	19	<0.001
Edema - 5 - Edema - 8 días	0.27	0.32	0.12	0.42	3.74	19	<0.001
Dolor - 3 - 5 días	1.25	1.33	0.63	1.87	4.19	19	<0.001
Dolor - 3 - 8 días	3.45	2.35	2.35	4.55	6.56	19	<0.001
Dolor - 5 - 8 días	2.20	1.79	1.36	3.04	5.48	19	<0.001
Trismus antes - Trismus 3 días	7.03	6.31	4.07	9.98	4.98	19	<0.001
Trismus antes - Trismus 5 días	5.94	5.86	3.20	8.68	4.54	19	<0.001
Trismus antes - Trismus 8 días	4.43	5.09	2.05	6.82	3.89	19	<0.001
Trismus 3 - Trismus 5 días	-1.08	1.70	-1.88	-0.28	-2.84	19	0.01
Trismus 3 - Trismus 8 días	-2.59	3.44	-4.20	-0.98	-3.37	19	<0.001
Trismus 5 - Trismus 8 días	-1.51	2.84	-2.84	-0.18	-2.38	19	0.03

Media. D. E.: desviación estándar. Mín.: mínimo y Máx.: máximo. t: total. gl: global

Prueba T de Student (p < 0.05)

Con la prueba estadística T de Student se identificó que existió una diferencia significa entre la media del edema y trismus en condiciones normales, en contraste con la obtenida a los 3, 5, 8 días al usar piezoeléctrico (p < 0,05); al igual que al comparar el dolor, trismus y edema posquirúrgico al tercer, quinto y octavo día (p < 0,05) (Tabla 4).

**Tabla 4:** Contraste de las variables iniciales y posquirúrgica con el uso del piezoeléctrico

Variables	Media	D. E.	95% IC		t	gl	p-valor
			Mín.	Máx.			
Edema - normal - 3 días	-0.46	0.41	-0.65	-0.27	-5.02	19	<0.001
Edema - normal 5 días	-0.27	0.34	-0.43	-0.11	-3.5	19	<0.001
Edema - normal 8 días	-0.12	0.26	-0.24	0.01	-1.98	19	0.06
Edema - 3 - 5 días	0.19	0.14	0.13	0.26	5.96	19	<0.001
Edema - 3 - 8 días	0.35	0.28	0.21	0.48	5.45	19	<0.001
Edema - 5 - 8 días	0.15	0.20	0.06	0.25	3.46	19	<0.001
Dolor - 3 - 5 días	2.44	2.23	1.34	3.55	4.65	17	<0.001
Dolor - 3 - 8 días	4.00	2.22	2.89	5.11	7.63	17	<0.001
Dolor - 5 - 8 días	1.40	2.48	0.24	2.56	2.53	19	0.02
Trismus antes - 3 días	14.26	10.59	9.30	19.22	6.02	19	<0.001
Trismus antes - 5 días	11.81	10.85	6.73	16.89	4.87	19	<0.001
Trismus antes - 8 días	7.81	9.82	3.21	12.40	3.55	19	<0.001
Trismus 3 - 5 días	-2.45	2.70	-3.71	-1.19	-4.06	19	<0.001
Trismus 3 - 8 días	-6.46	3.58	-8.13	-4.78	-8.07	19	<0.001
Trismus 5 - 8 días	-4.01	3.23	-5.51	-2.50	-5.55	19	<0.001
Tiempo de osteotomía total PM - Tiempo de osteotomía PME	-4.99	3.17	-6.47	-3.51	-7.04	19	<0.001

Media, D. E.: desviación estándar, Mín.: mínimo y Máx.: máximo, t: total, gl: global

Prueba T de Student (p < 0,05)

El menor tiempo en la osteotomía de los terceros molares inferiores retenidos del lado derecho (0,76 ± 0,27 min) e izquierdo (0,781 ± 0,33 min) es reportado por la pieza de mano recta rotatoria de baja velocidad, en comparación con el piezoeléctrico (derecho 3,31± 1,62 min; izquierdo 3,22 ± 1,55 min). Existió asociación entre el tiempo de osteotomía del lado derecho (p = 0,001) e izquierdo (p = 0,001) entre los dos instrumentos (p < 0,05) (Tabla 5).

**Tabla 5:** Tiempo de osteotomía con respecto a la pieza de mano recta y el piezoeléctrico

Variables	N	Pieza de mano recta rotatoria				Piezoeléctrico		p-valor	
		Media	D. E.	Mín.	Máx.	Media	D. E.	Mín.	Máx.	
Tiempo de osteotomía derecho	20	0.76	0.27	0.35	1.63	3.31	1.62	0.95	5.66	<0.001
Tiempo de osteotomía izquierdo	20	0.78	0.33	0.18	1.52	3.22	1.55	1.08	6.5	<0.001
Tiempo de osteotomía total	20	1.55	0.57	-	-	6.54	3.05	-	-	<0.001

N: número de pacientes. D. E.: desviación estándar. Mín.: mínimo y Máx.: máximo

Prueba T de Student (p < 0.05)

Además, se identificó que el menor tiempo total de osteotomía fue con la pieza de mano recta (1,55 ± 0,57 min), en comparación con el piezoeléctrico (6,54 ± 3,05 min). Existe una diferencia significativa (p = 0,001) entre la pieza de mano recta rotatoria y el piezoeléctrico (Tabla 5).

## DISCUSIÓN

La extracción quirúrgica de los terceros molares retenidos puede tener efectos secundarios posoperatorios como trismus, dolor, lesión nerviosa, inflamación y sangrados. Una posible solución es el cambio de estrategias que minimicen estas complicaciones; una de las opciones investigadas es la variación de la técnica de osteotomía o instrumental (rotatorio a piezoeléctrico) ^9^^,^^10^, debido a que estudios previos han reportado que los instrumentos de corte rotatorio presentan la desventaja de producir excesivo calor durante la osteotomía y aumenta la posibilidad de consecuencias antes descritas ^11^^,^^12^. Por lo antes expuesto, se determinó la eficacia del piezoeléctrico versus la pieza de mano recta a baja velocidad en los efectos posquirúrgicos (edema, dolor y trismus) y el tiempo de osteotomía, en la extracción de terceros molares inferiores retenidos de pacientes que acuden al área de Cirugía Maxilofacial del Hospital de Especialidades Fuerzas Armadas N.° 1, a fin de identificar cuál de los dos instrumentos rotatorios utilizados es el más efectivo.

El efecto posquirúrgico edema se presentó con mayor severidad a los 3 días con la pieza de mano recta a baja velocidad (el 40% de los casos fueron graves y el 35%, moderados) en contraste con el piezoeléctrico (el 65% fueron leves y el 25%, moderados). Esto es confirmado por el estudio de Basheer *et al*. ^11^ y Rajan *et al*. ^12^, quienes aducen que la extracción con piezoeléctrico produjo una respuesta inflamatoria menor, probablemente porque la vibración ultrasónica de la punta es menos dañina para el hueso; en cambio, con la rotatoria es mayor el edema por la acción térmica y el aumento de la respuesta inflamatoria al momento de la extracción de los terceros molares retenidos. Además, el piezoeléctrico evidencia mayor comodidad, menos vibraciones y ruido que pueden minimizar el estrés psicológico y la ansiedad del paciente.

Con ambas técnicas se demostró una disminución del edema con respecto al tiempo (3 días, 5 días y 8 días); sin embargo, a los 8 días, el 90% de los pacientes tratados con piezoeléctrico (11,37 mm) demostró un edema leve y con la pieza de mano recta fue un 75% leve (11,77 mm). Estos hallazgos fueron confirmados por Goyal *et al*. ^13^, en cuyo caso la inflamación posoperatoria estuvo presente en ambos grupos, con valores de mediciones significativamente mayores en el grupo pieza de mano recta (11,72 mm) que en el piezoeléctrico (11,27 mm), a los 8 días de la intervención, aunque determinaron que la inflamación se localizó en la región bucal en la mayoría de los casos. 

Al contrastar el edema causado por ambos grupos, se identificó una diferencia significativa a los 3, 5 y 8 días evaluados (p < 0,05). Estos hallazgos coinciden con diversos estudios, como los de Rajan *et al*. ^12^, Al-Delayme *et al*. ^14^ y Al-Moraissi *et al*. ^15^. No obstante, los resultados de la actual investigación difiere de Sivolella *et al*. ^16^, los cuales no encontraron diferencias estadísticamente significativas en la incidencia de limitaciones en la apertura de la boca, edema, signos clínicos de daño de tejidos blandos entre la pieza de mano recta y piezoeléctrico. Según Magesty *et al*. ^11^, el edema es una variable difícil de medir y puede haber un sesgo de medición, incluso con la calibración de los examinadores, ya que es una evaluación tridimensional, con recursos bidimensionales. Además, la literatura aduce que la extracción del tercer molar está influenciada por varios factores, como las angulaciones de la impactación, especialmente la distoangular, la extracción de hueso combinada con la sección del diente, la dificultad del procedimiento quirúrgico y la duración de la operación ^16^^,^^19^.

En cuanto al dolor, se reporta una mayor medición del dolor a los 3 días con la pieza de mano recta (4,80 ± 2,3) que el piezoeléctrico (4,66 ± 1,84); sin embargo, no existió una diferencia significativa al contrastar el dolor reportado por los pacientes por ambas técnicas a los 3 y 8 días (p > 0,05), similar situación a la que informa Jiang *et al*. _^10^_ , quienes utilizaron una escala analógica visual para identificar el grado de dolor en los pacientes expuesto a la extracción de terceros molares, estableciendo que el grupo con osteotomía con piezoeléctrico (experimental) sintieron menos dolor los primeros días posoperatorios, en comparación con el grupo con pieza de mano rotatorio (control), sin diferencias significativas en la puntuación del dolor entre los grupos experimental y de control. De acuerdo con Sivolella *et al*. ^16^, esto depende en gran medida de la técnica de colgajo empleada por el proceso de cicatrización, al igual que por la habilidad del operador. 

No obstante, se evidenció una mayor disminución significativa del dolor a los 5 días con el instrumento piezoeléctrico (p < 0,05), la escala del dolor para los pacientes tratados con pieza de mano recta fue de 3,55; en cambio, para el piezoeléctrico fue de 2,00. Sobre esto, Al-Delayme *et al*. ^14^ mencionan que el dolor fue más intenso y alcanzó su punto máximo durante el primer día posoperatorio con la pieza de mano rotatorio, con un variación significativa del dolor a los 5 días entre los grupos de estudio, el menor valor es del piezoeléctrico (1,39) en contraste con el rotatorio (2,26). Este comportamiento se atribuye al hecho de que la piezoeléctrica es mínimamente invasiva y óptima para proteger los tejidos blandos circundantes y estructuras importantes, como nervios, vasos, mucosas y huesos ^16^^,18^. 

Con respecto al trismus, se verificó que no existió diferencia significativa entre los grupos de piezoeléctrico y pieza de mano recta a los 3 y 5 días (p > 0,05), aunque los menores valores de apertura bucal fueron reportados por el piezoeléctrico. Estos hallazgos concuerdan con Menziletoglu *et al*. _^19^_ demostraron que no hubo diferencias significativas entre dos grupos (pieza de mano recta y piezoeléctrico) en el trismus posoperatorio, aducen que este comportamiento está relacionado con el factor edad de los pacientes, en vista que eran relativamente más jóvenes y las edades eran bastante cercanas entre sí, lo que probablemente aceleró el proceso de curación. Esta puede ser la explicación para los resultados del actual estudio, debido a que la mayoría de los participantes en ambos grupos tenían entre 18 y 21 años (80% pieza de mano recto y 70% piezoeléctrico). 

En cuanto a las variables demográficas, se comprobó que no existió diferencia significativa de las complicaciones (edema, dolor y trismus) entre el sexo y la edad de los pacientes con respecto a la osteotomía, dentro la exodoncia de terceros molares inferiores retenidos con piezoeléctrico y pieza de mano recta rotatoria a baja velocidad (p > 0,05). Esto se debe a que la mayoría de los pacientes en los diferentes grupos de estudio tenían entre 18 y 21 años, y el 55% de los pacientes de sexo femenino fue tratados con pieza de mano recta y 50%, con piezoeléctrico. La evidencia científica respalda estos resultados, como en el caso de Mantovani *et al*. _^18^_ , quienes no encontraron diferencias entre los dos grupos (piezoeléctrico y pieza de mano recta) respecto del dolor, edema y trismus reportados, considerando sexo y edad. 

Solo fue posible demostrar que en la pieza de mano recta existe diferencia significativa entre el dolor a los 3, 5 y 8 días con la edad (p < 0,05), en vista de que la mayoría de los pacientes afectados son jóvenes de 18 a 21 años con un nivel leve de dolor.

Otra variable analizada es el tiempo de la osteotomía, en la cual se identificó la diferencia significativa entre el tiempo de la extracción de los terceros molares retenidos (total, derecho e izquierdo) con el piezoeléctrico y la pieza de mano recta (p < 0,05), con duración en el procedimiento con piezoeléctrico (total = 6,54 ± 3,05 min), en contraste con la pieza de mano recta (total =1,55 ± 0,57 min). Estos resultados son confirmados por diversas investigaciones ^(15, 16, 19)^, como la de Al-Delayme *et al*. ^16^, quienes mencionan que la duración del procedimiento quirúrgico frente a las complicaciones posoperatorias es un tema controvertido. La cirugía piezoeléctrica requiere más tiempo en la osteotomía de terceros molares retenidos, en comparación con el instrumento rotatorio convencional, pero lo recomendable es que el tiempo de la cirugía no exceda los 30 minutos, debido a que la recuperación es más prolongada.

Sobre esto, Jiang *et al*. ^10^ señalan que la osteotomía con piezoeléctrico requiere más tiempo debido a la acción de corte micrométrico más lenta del dispositivo, lo que permiten un corte más selectivo de las estructuras óseas; esto reduce el daño tisular innecesario, lo que minimiza los episodios de dolor. Este lapso se reduce a medida que los especialistas adquieren más experiencia en la utilización del instrumental. 

Magesty *et al*. ^11^ expresan que el tiempo operatorio es directamente proporcional a la cantidad de inflamación provocada, lo cual se debe a que se requiere más tiempo para extraer un tercer molar, lo que provoca una mayor respuesta inflamatoria. Sin embargo, las cirugías realizadas con motores piezoeléctricos consumen más tiempo que con instrumentos rotativos comunes. Enfatizan que esta es una técnica relativamente nueva, por lo que la curva de aprendizaje puede haber sido fundamental para aumentar el tiempo del procedimiento, en comparación con los instrumentos rotativos. Asimismo, demuestran que el piezoeléctrico presenta los niveles más bajos de inflamación con respecto a hinchazón, dolor y trismus, resultados que concuerdan con los hallazgos del actual estudio y la aprobación de la hipótesis investigativa, donde los efectos posquirúrgicos (edema, dolor y trismus) de la exodoncia de los terceros molares inferiores retenidos se presentan en menor o igual intensidad con la utilización de piezoeléctrico en comparación con la pieza de mano recta rotatoria a baja velocidad.

Una de las limitaciones más importantes de la investigación es la disposición del instrumento piezoeléctrico para efectuar la osteotomía de los terceros molares inferiores retenidos, ya que este dispositivo es de alto costo y, por tanto, difícil de adquirir. Entre las fortalezas del estudio está dar a conocer la capacidad de aplicación clínica del piezoeléctrico para la extracción de terceros molares con mínimas complicaciones, por lo que sirve como una alternativa viable en contraste con la pieza de mano recta, además de identificar que es el sistema más eficaz para este tipo de cirugía.

Con los resultados de la investigación, se concluye que los efectos posquirúrgicos (edema, dolor y trismus) de la exodoncia de terceros molares inferiores retenidos, utilizando piezoeléctrico y pieza de mano recta rotatoria a baja velocidad, fueron más notorios en los jóvenes de 18 a 21 años de sexo femenino. El edema posterior a la exodoncia de terceros molares retenidos fue menor significativamente con el piezoeléctrico. La menor variación de la apertura bucal al tercer, quinto y octavo día de la exodoncia de terceros molares inferiores retenidos se produjo utilizando el piezoeléctrico, sin diferencia significativa del trismus entre la pieza de mano recta rotatoria a baja velocidad y el piezoeléctrico. El dolor posoperatorio fue menor en los pacientes que utilizaron el piezoeléctrico; sin embargo, solo existió una diferencia significativa al quinto día. Con respecto al tiempo de osteotomía, se identificó la relación entre el tiempo de la extracción de los terceros molares retenidos con el piezoeléctrico y la pieza de mano recta (p < 0,05), con mayor duración de la osteotomía al usar el piezoeléctrico (total = 6,54 min) en contraste con pieza de mano recta (total =1,55 min).

## Fuente de financiamiento:

rtículo autofinanciado por los autores
